# Rabies immunoglobulin utilization and delayed presentation following category III rabies exposure: a 10-year retrospective study in Hangzhou, China

**DOI:** 10.3389/fpubh.2026.1916685

**Published:** 2026-10-09

**Authors:** Jing Wang, Weiwei Cai, Jiaxin Chen, Jing Chen, Wanli Yang

**Affiliations:** 1 Department of Infectious Disease Control and Prevention, Hangzhou Center for Disease Control and Prevention (Hangzhou Health Supervision Institution), Hangzhou, China; 2 Department of Epidemiology and Biostatistics, School of Public Health and The Key Laboratory of Intelligent Preventive Medicine of Zhejiang Province, Zhejiang University School of Medicine, Hangzhou, China; 3 Department of General Medicine, Hangzhou Occupational Disease Prevention and Treatment Hospital, Hangzhou, China

**Keywords:** delayed presentation, post-exposure prophylaxis, rabies, rabies immunoglobulin, real-world study

## Abstract

**Background:**

Rabies is an almost invariably fatal, vaccine-preventable zoonosis. Rabies immunoglobulin (RIG) is recommended for Category III exposures, yet real-world RIG utilization remains poorly characterized. This study examined temporal trends and factors associated with RIG utilization and delayed presentation among Category III exposure patients in China.

**Methods:**

A retrospective study was conducted using clinical records of Category III exposure patients presenting to a high-volume rabies prevention clinic in Hangzhou, China, from 2015 to 2024. Calendar year 2018 was treated as a temporal reference year for descriptive comparison. Multivariable logistic regression identified factors associated with RIG non-administration and delayed presentation (>24 h post-exposure).

**Results:**

A total of 14,270 eligible cases were analyzed. Overall RIG utilization was 37.54%, declining from 53.09% in 2015 to 7.14% in 2022. RIG non-administration was independently associated with male sex (OR 1.20, 95% CI 1.11–1.29), age 18–59 years (OR 3.25, 95% CI 2.95–3.59), exposure at home (OR 1.47, 95% CI 1.33–1.63), presentation in 2018 (OR 2.38, 95% CI 2.01–2.82) and in 2019–2024 (OR 4.49, 95% CI 3.96–5.09), and delayed presentation (OR 1.43, 95% CI 1.32–1.55). The overall delayed presentation rate was 34.47%; independent risk factors included age 18–59 years (OR 1.37, 95% CI 1.24–1.52), exposure at home (OR 1.57, 95% CI 1.41–1.74), and presentation in 2018 (OR 1.61, 95% CI 1.40–1.86) and in 2019–2024 (OR 1.77, 95% CI 1.62–1.94).

**Conclusion:**

RIG utilization was low and showed marked temporal variation during 2015–2024, and one third of patients presented more than 24 h after exposure. Both outcomes were concentrated among working-age adults and domestic exposure settings. These findings describe service utilization patterns and identify subgroups in which gaps in PEP delivery are concentrated; targeted risk communication and routine monitoring of RIG utilization and presentation timeliness are needed.

## 1 Introduction

Rabies is an almost invariably fatal zoonosis caused by rabies virus infection, and no effective treatment exists once clinical symptoms appear (1, 2). The Global Burden of Disease Study 2019 estimated 14,075 new rabies cases and 13,743 associated deaths worldwide in that year, with a disproportionate burden in low- and middle-income countries (3). In regions where rabies is enzootic, domestic dogs serve as the principal reservoir, accounting for roughly 99% of human transmissions (4). China remains one of the countries with a high rabies burden: although officially reported human cases have declined markedly over the past two decades, rabies continues to rank among the leading infectious causes of death, particularly in rural areas and urban–rural fringe zones where animal bite incidents occur frequently (5–7). Tens of thousands of people in China receive rabies post-exposure prophylaxis (PEP) after animal bites each year, with rabies prevention clinics in Hangzhou alone treating tens of thousands of patients annually (8).

Because no effective treatment exists once clinical rabies develops, timely and standardized PEP remains the only effective preventive measure (1, 2). According to the World Health Organization (WHO) exposure classification, Category III exposures, including penetrating skin bites or scratches, licks on broken skin or mucous membranes, and bat exposures, require both rabies vaccination and rabies immunoglobulin (RIG) (1, 2). RIG provides immediate passive protection by neutralizing residual virus at the wound site during the 7 to 14 days before vaccine-induced active immunity develops, and is particularly important for patients with bites to nerve-rich areas such as the head, face, and hands, and for those with multiple or deep wounds (1, 2). The 2018 WHO position paper on rabies vaccines and China’s national regulations on rabies exposure prevention both recommend RIG administration for all Category III exposure patients without prior rabies vaccination, and RIG may be administered up to 7 days after the first vaccine dose (1, 9).

Despite these clear recommendations, real-world PEP delivery falls short of the guidelines in many settings. First, RIG non-administration is widespread: even among Category III exposure patients, reported utilization rates remain far below recommended levels (8–10). A previous study in Hangzhou found that although Category III exposures accounted for a considerable proportion of clinic visits, RIG utilization remained low (8), and inadequate wound treatment and delayed PEP are common among animal bite patients in China (10). Second, delayed presentation is another prominent problem. Many patients fail to seek care promptly after exposure, with reported reasons including insufficient awareness of rabies risk, neglect of minor wounds, financial barriers, and limited healthcare accessibility (10–12). A Tanzanian investigation found that a quarter of potentially exposed individuals did not attend medical facilities at all (11), and studies from Iran identified women and rural residents as more likely to delay care-seeking (12).

In addition, the relationship between exposure context, risk perception, and healthcare-seeking behavior has not been adequately studied. Existing research has focused mainly on individual-level factors affecting PEP compliance, while less attention has been paid to how exposure settings (domestic versus public places) and animal ownership jointly shape risk perception, the timing of care-seeking, and RIG acceptance (13, 14). Furthermore, the 2018 WHO position paper on rabies vaccines introduced important revisions concerning the administration and resource-efficient use of rabies immunoglobulin (RIG), while the indication for RIG in Category III exposures had already been established in earlier WHO recommendations (9). Whether RIG utilization patterns changed temporally around this period in China remains insufficiently characterized.

In this study, a management gap is operationalized as a population-level shortfall between guideline-recommended PEP and the care actually delivered, measured through two indicators: non-administration of RIG among Category III exposure patients without prior rabies vaccination, and presentation more than 24 h after exposure. Each reported variable serves a defined role in identifying these gaps: demographic and exposure-related variables characterize the subgroups in which gaps concentrate; the exposure-to-presentation interval captures the timeliness of care-seeking; and the study period variable, defined relative to the 2018 WHO position paper, captures system-level change in service delivery.

Using 10 years of clinical records from a high-volume rabies prevention clinic in Hangzhou, China, this study aimed to (1) describe temporal trends in RIG utilization from 2015 to 2024, (2) identify factors associated with RIG non-administration, (3) identify determinants of delayed presentation, and (4) generate evidence to inform targeted public health interventions.

## 2 Methods

### 2.1 Study design and setting

This retrospective observational study was conducted at a rabies vaccination clinic of Hangzhou Occupational Disease Prevention and Control Hospital in Hangzhou, China. Medical records from January 2015 to December 2024 were reviewed.

The study focused on patients with category III rabies exposure, representing the highest-risk group requiring comprehensive PEP.

### 2.2 Study population

The study population consisted of all patients diagnosed with Category III rabies exposure who sought medical care at the designated clinic during the study period. Eligible participants were required to meet the following inclusion criteria: confirmed category III rabies exposure and administration of the first dose of rabies vaccination at the study clinic after exposure. Individuals were excluded from the study if they had incomplete data on key variables (such as rabies immunoglobulin administration and presentation interval), possessed duplicate medical records, had a prior history of rabies vaccination, or underwent pre-exposure prophylaxis at other medical institutions before admission to the study clinic.

### 2.3 Data collection

Relevant data were retrospectively extracted from standardized clinical medical records of study participants. Variables collected fell into three predefined domains: demographic characteristics, exposure-related factors, and clinical management parameters. Demographic variables included age (categorized as <18, 18–59, and ≥60 years based on established epidemiological conventions), sex, and self-reported occupation. Exposure-related factors comprised animal type and exposure location (home, neighbor’s residence, public space, or other places). Clinical management parameters included time from exposure to first medical consultation (≤24 vs. >24 h), administration of rabies immunoglobulin (RIG) (yes/no).

Animal type was classified according to ownership/rabies risk as domestic animals, high-risk animals (including stray animals, wild animals, and suspected rabid animals), and animals with undetermined ownership.

### 2.4 Outcome definitions

The primary outcome was RIG non-administration, defined as the absence of RIG administration among patients with Category III rabies exposure and no prior rabies vaccination (1, 2, 9). Eligibility for this outcome was thus determined at the patient level using two variables recorded in the register: exposure category and vaccination history. RIG non-administration is used in this study as a population-level indicator of service utilization. It does not imply an assessment of the appropriateness of any individual treatment decision, because the register does not record the reasons for non-administration in individual cases, such as clinical judgment, patient refusal, product availability, or cost.

The secondary outcome was delayed presentation, defined as a first medical consultation more than 24 h after the rabies exposure. The 24-h threshold was selected because WHO and national guidelines recommend initiating PEP as early as possible after exposure, and presentation within 24 h is widely used as an indicator of timely care-seeking in the rabies literature (2, 15, 16). We acknowledge that the clinical benefit of PEP is not strictly limited to 24 h; this threshold is used here as a programmatic indicator of timely healthcare-seeking rather than a biological cutoff.

### 2.5 Time period definition

Because the WHO position paper was issued in April 2018, calendar year 2018 was treated as a transition year for descriptive temporal comparison rather than as a causal intervention point. The primary analysis compared the pre-2018 period (2015–2017) with the post-2018 period (2019–2024), with 2018 entered as a separate category in all regression models.

### 2.6 Statistical analysis

All categorical variables were described in terms of frequencies and relative percentages. Differences across groups were examined using the chi-square test or Fisher’s exact test, depending on the situation. To address potential imbalance in sample size across study periods, proportional data were analyzed, and study period (pre-2018, transition year 2018, post-2018) was incorporated as a three-category covariate in all multivariable models. Variables included in the multivariable logistic regression models were selected *a priori* based on their clinical and epidemiological relevance to RIG administration and healthcare-seeking behavior. Study period was included in all multivariable models to account for temporal variation. Odds ratios (ORs) and their corresponding 95% confidence intervals (CIs) were calculated and reported. A two-sided *p* value < 0.05 was regarded as statistically significant.

Potential multicollinearity among covariates was assessed using variance inflation factors (VIFs). Sensitivity analyses were performed by removing potentially collinear categorical variables, and changes in the ORs of the remaining variables were assessed.

Descriptive data were organized using Microsoft Excel (Microsoft Office 365), while statistical analysis was performed using R software (version 4.5.2).

### 2.7 Ethical considerations

This study was approved by the Institutional Review Board of the Ethics Committee of the Hangzhou Center for Disease Control and Prevention (Hangzhou Health Supervision Institution) (Approval No. 2026–46). Prior to analysis, all direct and indirect personal identifiers were systematically removed in accordance with established data governance protocols; the resulting dataset comprises fully anonymized data, de-identified in accordance with the Personal Information Protection Law of China (PIPL). All procedures were conducted in accordance with relevant ethical standards. Comprehensive declarations, including unblind institutional identities and administrative statements, are provided in the separate “Declarations” file.

## 3 Results

### 3.1 Characteristics of study population

A total of 14,270 eligible cases were enrolled in this study. By age group, the majority were adults aged 18–59 years (*n* = 10,188; 71.39%), followed by individuals under 18 years (*n* = 2,295; 16.08%) and those aged ≥60 years (*n* = 1,787; 12.52%). The sex distribution was nearly balanced, with males comprising 50.81% and females 49.19% of the cohort. Among self-reported occupational categories, workers constituted the largest group (*n* = 3,945; 27.65%), followed by students (*n* = 1,240; 8.69%) and community-dwelling children (*n* = 1,147; 8.04%).

Domestic animals caused most exposure cases, with a total of 10,951 cases accounting for 76.74%. High-risk animal exposures referred to bites and scratches inflicted by suspected rabid animals, wild animals and stray animals, which involved 1,603 cases and took up 11.23%. With regard to exposure location, the home was the most common setting (*n* = 11,058; 77.49%), followed by public spaces (*n* = 2,273; 15.93%) and neighbors’ residences (*n* = 645; 4.52%).

Post-exposure management revealed that 34.47% of patients (*n* = 4,919) sought medical care more than 24 h after injury, and 62.46% (*n* = 8,913) did not receive rabies immunoglobulin (RIG), despite guideline-recommended indications (see Table 1).

**Table 1:** Demographic and clinical characteristics of patients with Category III exposures in Hangzhou, China, 2015–2024.

Variable	Number (%)
Total	14,270
Age group (y)	
<18	2,295 (16.08)
18–59	10,188 (71.39)
≥60	1787 (12.52)
Sex	
Male	7,250 (50.81)
Female	7,020 (49.19)
Occupation	
Worker	3,945 (27.65)
Student	1,240 (8.69)
Community-dwelling children	1,147 (8.04)
Retired	1,111 (7.79)
Employee	1,010 (7.08)
Unemployed	197 (1.38)
Other	5,620(39.38)
Animal type	
Domestic	10,951 (76.74)
High-risk	1,603(11.23)
Undetermined ownership	1716 (12.03)
Exposure location	
Home	11,058 (77.49)
Public place	2,273 (15.93)
Neighbor	645 (4.52)
Other places	294 (2.06)
Delay of clinic presentation (>24 h)	
Yes	4,919 (34.47)
No	9,351 (65.53)
RIG use	
Yes	5,357 (37.54)
No	8,913 (62.46)

The annual number of eligible patients declined markedly over the study period, from 6,318 in 2015 to 3,422 in 2016, 1,300 in 2017, and 861 in 2018, and remained substantially lower thereafter, with 655 patients in 2019, 426 in 2020, 416 in 2021, 322 in 2022, 250 in 2023, and 300 in 2024, for a total of 14,270 eligible patients.

### 3.2 Utilization of rabies immunoglobulin

We retrospectively analyzed rabies immune globulin (RIG) administration among patients presenting with WHO-defined Category III exposures at this outpatient rabies prevention clinic from 2015 to 2024. RIG utilization hit its peak at 53.09% in 2015. It then declined steadily until 2022 and bottomed out at 7.14%, before rising to 21.00% in 2024 (Figure 1).

**Figure 1:**
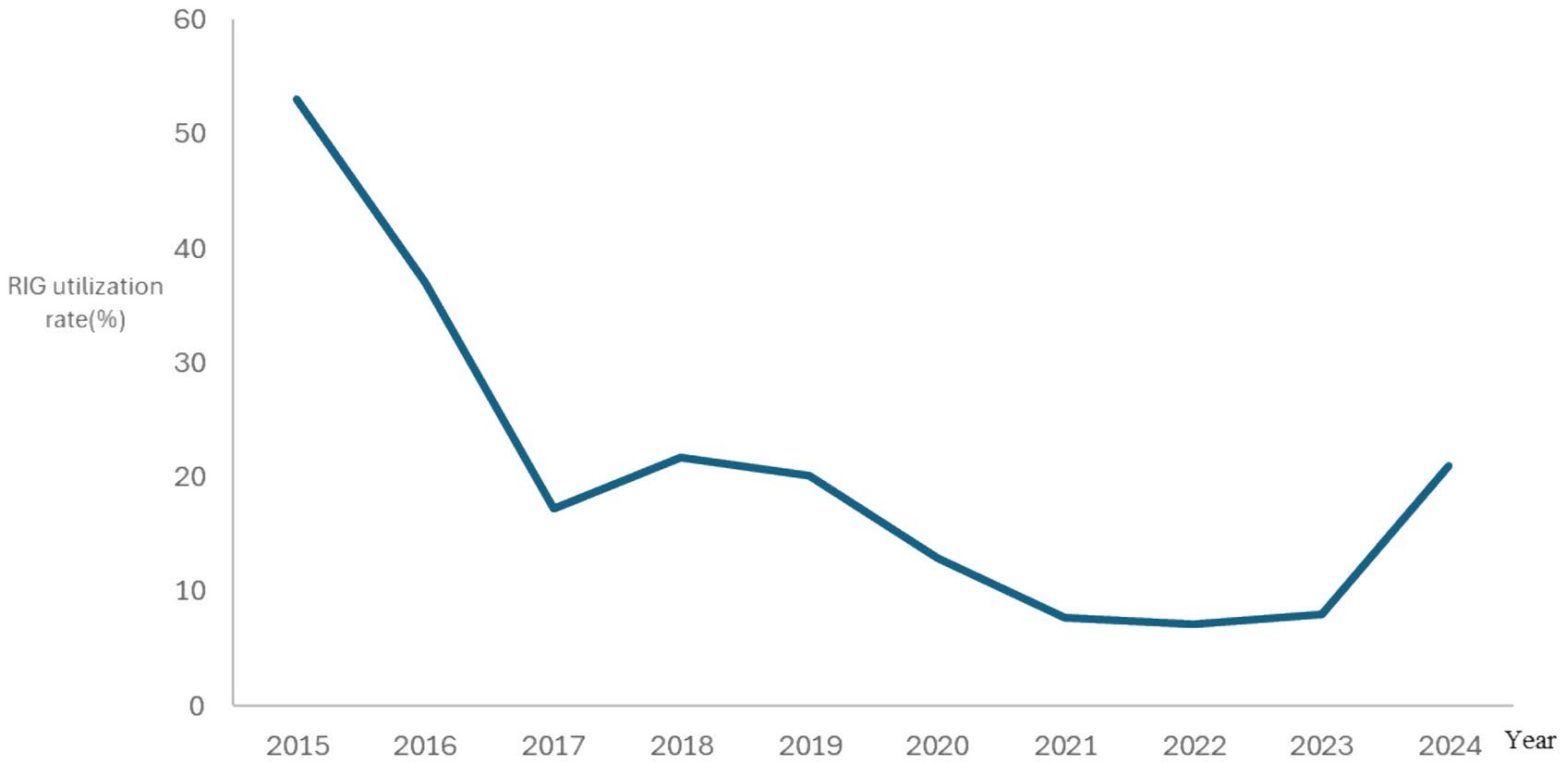
Temporal trends in rabies immunoglobulin (RIG) utilization rates among patients with WHO-defined Category III exposures, 2015–2024. The annual RIG utilization rate decreased from 53.09% in 2015 to a historic low of 7.14% in 2022, before rebounding to 21.00% in 2024.

In April 2018, the World Health Organization (WHO) issued an updated position paper on rabies vaccines that included important revisions concerning RIG administration and resource-efficient use. To characterize temporal differences in RIG utilization, we compared the pre-2018 period (2015–2017), the transition year (2018), and the post-2018 period (2019–2024). The RIG administration rate was 43.9% in 2015–2017, dropped to 21.7% during the 2018 transition year, and declined further to 13.7% in 2019–2024.

### 3.3 Univariate analysis of factors associated with RIG non-administration

We conducted univariate analysis on the use of immunoglobulin (RIG) among patients with Category III exposure seen at this outpatient clinic. Regarding age groups, the highest RIG usage rate was observed in patients under 18 years old, reaching 63.05%, while the group aged 18–59, despite having the largest number of cases, had the lowest RIG usage rate at 32.09%. There were statistically significant differences among the three age groups (*p* < 0.001). In terms of sex, female patients showed a slightly higher RIG usage rate than males (38.53% vs. 36.58%), which was also statistically significant (*p* = 0.017).

Half of the patients exposed to neighbors’ homes received RIG, and approximately 44% of those exposed in public places also used immunoglobulin, whereas this proportion dropped to 35.54% for patients exposed at home. There were statistically significant differences across exposure locations (*p* < 0.001). Patients injured by animals of unknown origin had the highest RIG usage rate at 38.11%, while those exposed to high-risk animals had the lowest at 35.06%. However, no significant difference was found within these groups (*p* = 0.091).

In terms of timing, patients exposed before 2018 had a significantly higher RIG usage rate compared to those exposed after 2018 (43.89% vs. 13.68%). Additionally, patients who sought care without delay had a higher RIG usage rate than those with delayed presentation (41.58% vs. 29.86%). Both comparisons were statistically significant (*p* < 0.001) (see Table 2).

**Table 2:** Univariate analysis of factors associated with rabies immunoglobulin (RIG) utilization among patients with Category III rabies exposure.

Variable	No. of RIG Use	No. of RIG Not Use	RIG Utilization Rate (%)	Chi-square	*p*-value
Age group				768.29	<0.001
<18	1,447	848	63.05		
18–59	3,269	6,919	32.09		
≥60	641	1,146	35.87		
Sex				5.72	0.017
Male	2,652	4,598	36.58		
Female	2,705	4,315	38.53		
Exposure location				104.62	<0.001
Home	3,930	7,128	35.54		
Neighbor	323	322	50.08		
Public place	1,002	1,271	44.08		
Other places	102	192	34.69		
Animal type				4.80	0.091
Domestic	4,141	6,810	37.81		
High-risk	562	1,041	35.06		
Undetermined ownership	654	1,062	38.11		
Time				857.41	<0.001
Pre-2018	4,846	6,194	43.89		
Transition (2018)	187	674	21.72		
Post-2018	324	2045	13.68		
Time of presentation				188.16	<0.001
Timely presentation (≤24 h)	3,888	5,463	41.58		
Delayed presentation (>24 h)	1,469	3,450	29.86		

### 3.4 Multivariable analysis of factors associated with RIG non-administration

The multivariate logistic regression analysis revealed that factors influencing RIG use included male sex (Odds Ratio (OR) 1.199, 95% confidence interval (CI) 1.115–1.289), age group 18–59 years (OR 3.254, 95% CI 2.948–3.590), age group over 60 years (OR 2.895, 95% CI 2.532–3.309), exposure at home (OR 1.473, 95% CI 1.331–1.630), presence of domestic animals (OR 0.853, 95% CI 0.756–0.962), seeking medical care in year 2018 (OR 2.379, 95% CI 2.009–2.818) and after year 2018 (OR 4.489, 95% CI 3.959–5.089), as well as seeking care more than 24 h after exposure (OR 1.429, 95% CI 1.322–1.546) (see Table 3).

**Table 3:** Multivariable logistic regression analysis of independent risk factors associated with rabies immunoglobulin (RIG) non-administration.

Variable	Category	Reference	*B*	SE	*Z*	*p*	OR	95%CI
Sex	Male	Female	0.182	0.037	4.911	<0.001	1.199	1.115–1.289
Age group (y)	18–59	<18	1.180	0.050	23.470	<0.001	3.254	2.948–3.590
	>60	<18	1.063	0.068	15.569	<0.001	2.895	2.532–3.309
Exposure location	Home	Public place	0.387	0.052	7.475	<0.001	1.473	1.331–1.630
	Neighbor	Public place	−0.038	0.098	−0.388	0.698	0.963	0.795–1.166
	Other places	Public place	0.236	0.140	1.690	0.091	1.266	0.963–1.665
Animal type	Domestic	High-risk	−0.160	0.062	−2.590	0.010	0.853	0.756–0.962
	Undetermined ownership	High-risk	−0.076	0.078	−0.970	0.332	0.927	0.796–1.080
Time period	Transition (2018)	Pre-2018	0.867	0.086	10.039	<0.001	2.379	2.009–2.818
	Post-2018	Pre-2018	1.502	0.064	23.438	<0.001	4.489	3.959–5.089
Delay presentation	>24 h	≤24 h	0.357	0.040	8.929	<0.001	1.429	1.322–1.546

RIG, rabies immunoglobulin; OR, odds ratio; CI, confidence interval.

The maximum VIF was 6.05, with a mean VIF of 2.46 in the RIG non-administration model. Sensitivity analyses showed that removing the potentially collinear categorical variables resulted in less than 10% change in the ORs of the remaining variables, indicating that potential multicollinearity did not materially affect the main findings.

### 3.5 Univariate analysis of factors associated with delayed presentation

We performed univariate analysis of delayed presentation, which refers to seeking medical care more than 24 h after exposure, among patients with WHO-defined Category III exposures attending this outpatient rabies prevention clinic. Across age groups, the 18–59 years cohort exhibited the highest proportion of delayed presentation (37.70%), whereas patients aged ≥60 years had the lowest (23.28%). Significant intergroup differences were observed across all three age categories (*p* < 0.001). Although females demonstrated a marginally higher delay rate than males (35.07% vs. 33.89%), this difference was not statistically significant (*p* = 0.142).

By exposure location, patients exposed at neighbors’ residences had the lowest delay rate (26.36%), followed closely by those exposed in public places (27.67%). In contrast, exposures occurring at workplaces or other non-residential settings were associated with markedly elevated delay rates (38.78%). There were highly significant differences in delayed presentation across exposure locations (*p* < 0.001). With regard to animal classification, injuries may have contributed high-risk animals (e.g., stray or unvaccinated dogs/cats exhibiting abnormal behavior) were associated with the highest delay rate (36.81%), whereas exposures involving animals of unknown ownership were linked to the timeliest care-seeking behavior (delay rate: 28.73%). These differences were statistically significant (*p* < 0.001).

Temporal analysis revealed an increase in delayed presentation from 31.39% in the pre-2018 period (2015–2017) to 43.55% in year 2018, and to 45.50% in the post-2018 period (2019–2024), a change that was highly statistically significant (*p* < 0.001). All results are presented in Table 4.

**Table 4:** Univariate analysis of factors associated with delayed clinic presentation (>24 h) among patients with Category III rabies exposure.

Variable	No. of delayed presentation	No. of non-delayed presentation	Rate of delayed presentation (%)	Chi-square	*p*-value
Age group				178.31	<0.001
<18	662	1,633	28.85		
18–59	3,841	6,347	37.70		
≥60	416	1,371	23.28		
Sex				2.15	0.142
Male	2,457	4,793	33.89		
Female	2,462	4,558	35.07		
Exposure location				82.82	<0.001
Home	4,006	7,052	36.23		
Neighbor	170	475	26.36		
Public place	629	1,644	27.67		
Other places	114	180	38.78		
Animal type				30.42	<0.001
Domestic	3,836	7,115	35.03		
High-risk	590	1,013	36.81		
Undetermined ownership	493	1,223	28.73		
Time period				205.37	<0.001
Pre-2018	3,466	7,574	31.39		
Transition (2018)	375	486	43.55		
Post-2018	1,078	1,291	45.50		

### 3.6 Multivariable analysis of factors associated with delayed presentation

The multivariable logistic regression analysis revealed that factors associated with delayed medical visits included the age group of 18–59 years (OR 1.373, 95% CI 1.241–1.518), the age group over 60 years (OR 0.680, 95% CI 0.588–0.785), exposure at home (OR 1.565, 95% CI 1.408–1.739), exposure at workplaces or other locations (OR 1.595, 95% CI 1.232–2.065), owning household pets (OR 0.819, 95% CI 0.729–0.920), animals whose owners could not be identified (OR 0.645, 95% CI 0.554–0.751), and seeking medical care in year 2018 (OR 1.614, 95% CI 1.399–1.861) and after 2018 (OR 1.773, 95% CI 1.618–1.943) (see Table 5).

**Table 5:** Multivariable logistic regression analysis of predictors for delayed clinic presentation (>24 h) post-exposure.

Variable	Category	Reference	*B*	SE	*Z*	*p*	OR	95%CI
Age group (y)	18–59	<18	0.317	0.051	6.175	<0.001	1.373	1.241–1.518
	>60	<18	−0.386	0.074	−5.254	<0.001	0.680	0.588–0.785
Exposure location	Home	Public place	0.448	0.054	8.296	<0.001	1.565	1.408–1.739
	Neighbor	Public place	0.106	0.104	1.013	0.311	1.112	0.906–1.364
	Other places	Public place	0.467	0.132	3.547	<0.001	1.595	1.232–2.065
Animal type	Domestic	High-risk	−0.200	0.059	−3.379	<0.001	0.819	0.729–0.920
	Undetermined ownership	High-risk	−0.438	0.077	−5.671	<0.001	0.645	0.554–0.751
Time period	Transition (2018)	Pre-2018	0.478	0.073	6.580	<0.001	1.614	1.399–1.861
	Post-2018	Pre-2018	0.573	0.047	12.251	<0.001	1.773	1.618–1.943

RIG, rabies immunoglobulin; OR, odds ratio; CI, confidence interval.

The maximum was 5.83, with a mean VIF of 2.56. Sensitivity analyses showed that removing the potentially collinear categorical variables resulted in less than 10% change in the ORs of the remaining variables, indicating that potential multicollinearity did not materially affect the main findings.

## 4 Discussion

This study found that RIG utilization was 37.54% overall and showed marked temporal variation during 2015–2024. The delayed presentation rate was 34.47%, and delayed presentation was associated with RIG non-administration. These findings provide real-world evidence on temporal and contextual patterns in rabies PEP service utilization and identify subgroups in which gaps in PEP delivery are concentrated.

One of the most important findings of this study is the significant impact of exposure setting on the timing of medical visits and RIG utilization. Our results show that patients with domestic exposure not only had a higher likelihood of delayed presentation but also had significantly lower RIG utilization rates compared to patients with public place exposure. One possible explanation is “familiarity bias,” whereby patients may perceive animals they raise or know well as safe and underestimate the risk of rabies exposure (16). Because risk perception was not directly measured in this study, this interpretation remains hypothetical. This finding is consistent with previous studies. Liu et al. (16) pointed out that the probability of delayed PEP was higher after bites from owned animals, because patients believed that familiar animals were unlikely to carry the rabies virus. Similarly, El Bazi et al. (17) in Morocco found that patients were more likely to seek medical care promptly after stray animal bites, while bites from owned animals were more likely to be neglected. From a public health perspective, this highlights a critical gap in community-based risk communication, where overlapping management of domestic pets fails to translate into standard clinical care-seeking behavior.

In contrast to the familiarity bias, our study found that patients exposed to high-risk animals (such as stray animals or suspected rabid animals) had less delayed presentation. This pattern is consistent with the hypothesis that perceived risk influences healthcare-seeking behavior, although this mechanism could not be verified directly in our data. When patients realize they have been exposed to high-risk animals, the awareness of the severity of rabies (perceived severity) prompts them to seek medical help promptly (18). This finding is consistent with behavioral decision theory, which states that individual perception of risk severity directly affects health behavior decisions. Wilde et al. also pointed out that patients’ awareness of rabies fatality affects their PEP adherence (19). A KAP assessment conducted in Uganda demonstrated that individuals with adequate rabies knowledge had a higher probability of seeking medical help and receiving post-exposure prophylaxis after being exposed (20). Studies in Ethiopia similarly showed that although rabies knowledge awareness is high, there are still gaps in actual healthcare-seeking behavior (21). This underscores the need for public health interventions to focus not only on the severity of rabies but also on rectifying the common misperceptions surrounding low-risk-appearing clinical exposures.

Additionally, our study found that adults were more likely to delay presentation than children, which may be related to adults’ greater confidence in self-treating minor wounds, while child exposures are usually discovered by parents and treated promptly (22). This is consistent with previous findings that patients aged 15–44 years are more likely to delay PEP (16). Compared with children, working-age adults may face additional barriers to timely healthcare seeking, including occupational responsibilities, limited flexibility during working hours, and competing daily commitments. In addition, rabies prevention campaigns are often designed around children, families, and high-risk groups, whereas working-age adults may receive relatively less targeted health education, potentially contributing to insufficient risk perception.

Studies in Tanzania also found that the main reasons for delayed presentation were lack of awareness of rabies risk (approximately 25%) and economic barriers (10). Another possible explanation for lower RIG acceptance among working-age adults is the financial burden associated with RIG. Because RIG remains a relatively expensive component of PEP, and financial barriers have been reported as obstacles to rabies prevention in different settings (10, 11), financial considerations may influence patients’ decisions regarding RIG administration. However, information on income level and health insurance status was unavailable in our database, and the potential contribution of socioeconomic factors requires confirmation in future studies. Overall, the increased risk of delayed presentation and RIG non-administration among working-age adults may reflect a combination of behavioral, socioeconomic, and healthcare-seeking behavior-related factors rather than a single age-related effect.

The transition-year analysis suggests that this shift was gradual rather than abrupt: the odds of RIG non-administration were already elevated in 2018 (OR 2.38 vs. 2015–2017) and increased further in 2019–2024 (OR 4.49). The temporal pattern observed in this study should be interpreted cautiously. Although 2018 coincided with the publication of an updated WHO position paper that included important revisions concerning RIG administration and resource-efficient use, the decline in RIG utilization observed in our study had already begun before 2018. In particular, the number of eligible patients decreased substantially from 6,318 in 2015 to 3,422 in 2016 and 1,300 in 2017, before the 2018 transition year. Therefore, our findings do not establish that the 2018 WHO position paper caused the subsequent decline in RIG utilization. The substantial reduction in the number of eligible patients over the study period also warrants consideration when interpreting the observed temporal patterns. During the study period, the availability of rabies post-exposure prophylaxis services in Hangzhou increased, and the catchment population and referral pathways of individual clinics may therefore have changed over time. Consequently, the population attending the study clinic may not have remained constant throughout the 10-year period. Although detailed annual information on referral pathways and catchment-area changes was unavailable, changes in the underlying patient population may have contributed to the observed temporal differences and represent a potential source of selection bias. Accordingly, the observed post-2018 pattern should be regarded as a temporal association rather than evidence of a causal effect of the 2018 WHO position paper.

Previous studies have shown that shortages, high costs, and restricted availability of RIG remain common barriers to optimal PEP implementation worldwide (10, 23, 24). Under such circumstances, clinicians may rely more heavily on careful risk assessment when determining the need for RIG (1, 25). Therefore, the observed variation in RIG utilization may reflect a combination of local healthcare system changes, patient and provider factors, and real-world constraints in PEP delivery. However, the relative contribution of these factors could not be determined from our data.

Notably, studies from the Netherlands showed that after the 2018 WHO guideline update, the local infiltration strategy for RIG could significantly conserve RIG usage (59% savings) without compromising protective efficacy (26). Real-world evidence from Serbia also showed a similar trend, where HRIG use was associated with lower rabies virus neutralizing antibody (RVNA) titers after the 4-dose vaccination regimen, suggesting that clinicians may need to reconsider RIG use strategies (27). These findings illustrate that RIG administration practices have evolved in different settings, but they do not establish that the temporal pattern observed in our study was caused by the 2018 WHO update.

Our study found a significant association between delayed presentation and RIG non-administration, with RIG utilization rate of only 29.86% among delayed presenters, far lower than timely presenters. Several possible mechanisms may contribute to this observed association. However, these factors were not directly measured in the present study, and their individual contributions cannot be determined from the available data.

Notably, even among patients presenting within 24 h of exposure, in whom RIG would almost invariably be indicated, the utilization rate was only 41.58%, suggesting that non-administration cannot be attributed solely to delayed presentation or wound healing status. First, from a medical perspective, delayed presentation may reduce the opportunity for timely RIG administration. WHO recommends that RIG should be used as soon as possible after exposure (1), because RIG provides passive immune protection before vaccine-induced active immunity develops (typically requiring 7–14 days), neutralizing residual virus at the wound site. However, delayed presentation does not necessarily eliminate the potential benefit of RIG, and clinicians should consider exposure characteristics, wound assessment, and timing of presentation when evaluating the need for RIG.

Second, from the perspective of patient behavior, patients with delayed presentation may have lower risk perception of rabies, and this low risk perception not only leads to delayed presentation but also affects patients’ willingness to accept RIG. Kumar et al. in New Delhi, India found that the main barriers to delayed presentation included lack of awareness (15.5%), distance to healthcare facilities (23.7%), and work constraints (23.9%) (15). Some patients may believe that since presentation is already delayed, the necessity of RIG is also reduced (16). Therefore, delayed presentation may represent a marker of insufficient rabies risk perception rather than an independent cause of RIG non-administration.

Third, from the clinical practice perspective, wound healing status after delayed presentation may influence clinicians’ assessment regarding RIG administration. However, such decision-making processes were not captured in our database. Given that the rabies incubation period can extend from several weeks to several months (averaging 1–3 months), the need for RIG should not be determined solely based on wound healing status (28). Future studies incorporating both patient and provider perspectives are needed to clarify the mechanisms underlying this association.

Our study found that patients younger than 18-years old had more standardized PEP practices, reflected in the higher RIG utilization rate compared to adults. This difference may be related to several factors. For one thing, parents generally pay more attention to children’s health than to their own. When children suffer animal bites, parents often exhibit higher levels of anxiety, which prompts them to more actively seek medical help and complete the PEP regimen (22). This is consistent with previous findings that parents are more sensitive to young people exposure. Parents usually have a clearer understanding of the severity of rabies, especially those who have learned through health education that rabies has a 100% fatality rate, and they are more likely to actively seek PEP for their children (29). For another, from the clinical practice perspective, clinicians tend to be more cautious in managing pediatric exposures. In addition, children are more frequently bitten on the head, face, and hands, which together account for approximately 40% of bites among young patients (30). Because RIG indication is determined by exposure category and prior vaccination history, clinicians may be more inclined to administer RIG promptly for such anatomically severe exposures. Studies in the UAE showed that 84% of pediatric patients received rabies vaccination, reflecting the high priority given to pediatric exposures (30).

Interestingly, in the multivariate logistic regression analysis, the independent effect of animal type was diminished. This suggests that healthcare-seeking behavior and RIG utilization may be more influenced by exposure context (contextual factors) rather than animal category itself. It should be noted that animal type in this study was categorized according to ownership/rabies risk rather than species.

This finding supports the concept of “contextual risk perception,” whereby patients’ judgment of rabies risk is not only based on animal species but also influenced by the environment and context of exposure (13). For example, regardless of animal species, exposures occurring in domestic settings may trigger “familiarity bias”; while exposures encountered in public places, whether from stray animals or not, may be perceived as higher risk (17). Studies in Iran also found that rural residents and women were more likely to delay presentation, indicating that socioeconomic factors and geographic location are also important influencing factors (11). Studies in Pakistan showed that in rural areas, delayed presentation after rabies exposure is more common due to inadequate medical facilities and cultural factors (12).

Additionally, our findings are consistent with the theory of behavioral determinants. Previous studies have shown that factors such as exposure context, timing of healthcare-seeking, and risk perception may better predict PEP completeness and standardization than animal classification (15, 16). This suggests that public health interventions may need to focus more on contextual factors rather than simply emphasizing the danger of specific animal species.

The data yielded by our investigation holds important practical value for the development of targeted rabies prevention and control programs. First, health education should focus specifically on “seemingly healthy” animals and owned animal exposures. Our study shows that these seemingly “low-risk” exposures actually lead to higher rates of delayed presentation and RIG non-administration. Health education programs should emphasize the key message that “apparently healthy owned animals may also carry the rabies virus” (29). Studies in Kenya proved that SMS reminders can significantly improve PEP adherence, providing new ideas for improving health education methods (31). Second, targeted education is needed for adult populations. Our study found that adults are more likely to delay presentation, which may be related to adults’ tendency to self-treat wounds. Public health interventions should specifically emphasize that adults should also seek medical attention promptly and not delay treatment simply because of “just a small wound” (16). Third, educational content should emphasize risk assessment rather than focusing solely on animal species. Patients need to understand that factors such as exposure context (e.g., at home, with familiar animals) and post-exposure behaviors (e.g., delayed presentation) affect risk, rather than judging solely based on whether the animal “looks normal” (17).

Some limitations of this study warrant acknowledgment. First, as a retrospective analysis of a clinic register, the study contained no post-exposure follow-up information. Whether any patient subsequently developed rabies could not be ascertained from our data source, and no PEP failure outcome was defined. Our findings therefore do not address the clinical effectiveness of PEP or the consequences of RIG omission. Second, the register was designed for epidemiological recording and did not capture the details of PEP delivery, including the vaccine product and manufacturer, vaccination route and schedule, adherence to and completion of the prescribed course, RIG type, potency, calculated dose, or wound infiltration technique. Consequently, we could not adjudicate the appropriateness of individual treatment decisions, nor distinguish whether non-administration in a given case reflected clinical judgment, patient refusal, product availability, or cost. The primary outcome should accordingly be interpreted as a service-utilization indicator rather than a measure of clinical appropriateness. It is nonetheless worth noting that RIG utilization was only 41.58% even among patients presenting within 24 h of exposure, in whom RIG would almost invariably be indicated, which suggests that the low utilization observed is unlikely to be fully explained by appropriate clinical discretion. In addition, wound severity, patient-reported risk perception, reasons for RIG refusal, and clinicians’ decision-making processes were not captured; the mechanisms underlying the observed associations therefore remain speculative and should be tested in prospective studies incorporating patient and provider perspectives. Third, the single-center design limits the generalizability of the results to other regions of China, given regional differences in rabies prevalence and healthcare resources (6). Fourth, selection bias related to changes in the study clinic population cannot be excluded. The number of eligible patients declined substantially over the study period, and the increasing availability of rabies PEP services in Hangzhou may have altered referral pathways and the catchment population of the study clinic. Detailed annual information on referral patterns and catchment-area changes was unavailable. Therefore, temporal differences in RIG utilization may partly reflect changes in the underlying patient population rather than changes in clinical practice alone. In addition, information bias inherent to retrospective register-based studies may have affected the accuracy of the recorded variables (28). Finally, socioeconomic variables such as income and health insurance coverage were unavailable, and residual confounding therefore cannot be excluded.

Despite the aforementioned limitations, this research provides credible real-world evidence to identify key problems in domestic rabies PEP practice. Future research could consider multi-center designs and prospective cohort studies to further validate our findings and explore the underlying reasons for RIG non-administration.

## 5 Conclusion

In this 10-year retrospective study of 14,270 Category III exposure patients in Hangzhou, China, 37.54% received rabies immunoglobulin, and RIG utilization showed marked temporal variation during 2015–2024. One third of patients presented more than 24 h after exposure. RIG non-administration and delayed presentation were more frequent among working-age adults and patients exposed in domestic settings. Because individual treatment details, reasons for RIG non-administration, and follow-up information were unavailable, these findings describe service-utilization patterns rather than the appropriateness or clinical outcomes of individual care. Targeted risk communication and continued monitoring of RIG utilization and presentation timeliness may help improve PEP service delivery and support the WHO “Zero by 30” goal.

## Data Availability

The raw data supporting the conclusions of this article will be made available by the authors, without undue reservation.
